# Teaching the health advocacy role in family medicine: Trial and error

**DOI:** 10.1080/13814788.2019.1674048

**Published:** 2019-10-16

**Authors:** Peter Decat, Meral Demirören, An De Sutter

**Affiliations:** Department of Public Health and Primary Care, Faculty of Medicine and Health Sciences, Ghent University, Ghent, Belgium; Department of Medical Education and Informatics, Hacettepe University Faculty of Medicine, Ankara, Turkey

September, the start of the postgraduate training at the faculty of medicine. Traditionally, two topics dominate the introduction for students in family medicine: the warm days of the inevitable Indian summer and the seven canmeds roles that describe the abilities physicians should have to meet the health care needs of patients. As experienced lecturers, we do not hesitate in telling the students in detail how to act as medical expert, communicator, collaborator, scholar, manager or professional. However, there is one of the roles that we keep for the end, hoping to be saved by the bell, or that we, accidentally or intentionally, forget: the family physician as a health advocate. The role of health advocacy is seen as one of the most challenging roles to teach and evaluate [1]. Poulton and Rose identified three key barriers affecting health advocacy education: a lack of clarity of the concept, insufficient experience in how to put health advocacy learning in practice and nonexistence of a standard for assessment [2].

First, there is a need for a good definition of the concept. Is the health advocacy role limited to the interaction between the physician and the individual patient? Or does it also entail an engagement beyond the practice walls? We believe that family doctors should be trained in the preparedness and skills to promote health at individual, community and policy level [3]. Recently, the Centre of Family Medicine University Ghent added to their mission statement that they have a particular interest for the social accountability of family doctors as activists for universal access to health care and as signallers of social injustice and exclusion. This is in line with the modified definition of the health advocate role within the CanMeds framework that states: ‘physicians contribute their expertise and influence as they work with communities or patient populations to improve health. They work with those they serve to determine and understand needs, speak on behalf of others when required, and support the mobilization of resources to effect change’ [4].

But how to teach this? Although, difficult and with little agreement on how it should be done, we developed at our university a programme to make students more aware of their future health advocacy role. The aim is to introduce them directly to clinical and nonclinical settings that work for and with vulnerable populations. In this way, students, often themselves belonging to a higher socio-economic class, are exposed to inequity, poverty and ill-making contexts and find out how as future family doctors they may contribute positively to the health of individuals and communities in cooperation with other professionals, welfare organisations, volunteers and patients. This exposure happens through participation in various local initiatives supported by the city governance, community health centres or welfare organisations. Here, we will briefly present some examples. A first example is a pilot project with community health workers, funded by the city council. The initiative aimed to bridge the gap between disadvantaged groups and the official healthcare system. Medical students actively participated in the training of community health workers and others were involved in the evaluation of the project. In another project, students collaborated with teachers of primary schools in deprived areas for lessons on mental health and nutrition. In a third project, a card play was designed and tested to be used as a tool to discuss sexual rights among young migrant women. Further, a group of students conducted focus group discussions and interviews with community members to explore the accessibility of health care services in the city. Again, others participated in various community-based activities promoting sexual health, healthy feeding, mental health or physical exercise.

The communities and the students highly appreciate these practical experiences. Students recognise that this exposure contributes to their awareness of the needs of this vulnerable population. Besides, it brings them in contact with role models in health care and welfare that shape their perspectives. The feeling of making a difference makes them feel fulfilled and creates motivation to provide responsive care to the needs of communities and individuals. Finally, it contributes positively to the image of family doctors as advocates of health in and with the community.

Place-based learning through participation at health-promoting and aware making activities contributes to students competences to respond to health needs at the individual and community level. However, there is still the policy part of the health advocacy role. As family physicians, we operate from within the society and are well placed to bring health issues at the political level. We do not plead to include policy training in the standard package for all postgraduates in family medicine. However, some students have the interest and the personal characteristics to act at the political level. These students need to be identified and to be supported in their development as change-makers.

Finally, standards for evaluating the health advocacy role are lacking. There is a need for tools that measure the impact of training. And what aspects should be tested? Knowledge on social determinants and the readiness to integrate the advocacy role in professional practice are fundamental [5,6]. Therefore, Belgian and Turkish medical institutes will develop and assess an instrument to scale this knowledge and motivation among medical students.

As medical institutes, we have the mission to deliver social accountable physicians. Future family physicians who will work with and in communities, should be prepared to advocate health at individual, community and political level. There are no standards on how this can be taught. It is a matter of trial and error. To our experience place-based learning is an excellent mechanism to shape awareness and motivation among students. In a next step, we will try out strategies for the evaluation of the health advocacy role and for the support of students and physicians who feel called upon to voice health needs at the policy level.
